# PDE5 inhibitors enhance the lethality of [pemetrexed + sorafenib]

**DOI:** 10.18632/oncotarget.14562

**Published:** 2017-01-09

**Authors:** Laurence Booth, Jane L. Roberts, Andrew Poklepovic, Paul Dent

**Affiliations:** ^1^ Department of Biochemistry and Molecular Biology, Virginia Commonwealth University, Richmond, VA 23298-0035, USA; ^2^ Department of Biochemistry and Medicine, Virginia Commonwealth University, Richmond, VA 23298-0035, USA

**Keywords:** sildenafil, autophagy, pemetrexed, chaperone, lung cancer

## Abstract

The combination of pemetrexed and sorafenib has significant clinical activity against a wide variety of tumor types in patients and the present studies were performed to determine whether sildenafil enhances the killing potential of [pemetrexed + sorafenib]. In multiple genetically diverse lung cancer cell lines, sildenafil enhanced the lethality of [pemetrexed + sorafenib]. The three-drug combination reduced the activities of AKT, mTOR and STAT transcription factors; increased the activities of eIF2α and ULK-1; lowered the expression of MCL-1, BCL-XL, thioredoxin and SOD2; and increased the expression of Beclin1. Enhanced cell killing by sildenafil was blocked by inhibition of death receptor signaling and autophagosome formation. Enforced activation of STAT3 and AKT or inhibition of JNK significantly reduced cell killing. The enhanced cell killing caused by sildenafil was more reliant on increased PKG signaling than on the generation of nitric oxide. *In vivo* sildenafil enhanced the anti-tumor properties of [pemetrexed + sorafenib]. Based on our data we argue that additional clinical studies combining pemetrexed, sorafenib and sildenafil are warranted.

## INTRODUCTION

Sorafenib, in addition to being an inhibitor of protein ser/thr/tyr kinases was also recently discovered to be a potent inhibitor of chaperone ATPase activities that are associated with conformational changes in the ATP binding NH_2_-termini of the chaperone proteins and with the abilities of these chaperones to associate and co-localize with other chaperones as well as client proteins [1, 2]. It has been reported by others that cGMP/PKG phosphorylation of chaperones inactivates their ATPase activities [3, 4]. We recently determined that that PDE5 inhibitors such as sildenafil (Viagra^®^) did not significantly alter basal chaperone ATPase activities but instead facilitated the drugs sorafenib, regorafenib and pazopanib to cause further inhibition of chaperone ATPase activities and a more rapid NH_2_-terminus conformational change which was reliant on PKG activation [1, 2, 5–9]. In addition, the thymidylate synthase inhibitor pemetrexed enhanced the ability of sorafenib to reduce GRP78 chaperone activity and increase eIF2α phosphorylation [2]. In the recently published companion paper to this manuscript we demonstrated that pemetrexed could also reduce chaperone activity, that required basal levels of PKG phosphorylation [10].

The changes in chaperone ATPase activity and chaperone conformation after [sorafenib + pemetrexed], [regorafenib + sildenafil], [sorafenib + sildenafil] and [pazopanib + sildenafil] exposure were also reflected in the phosphorylation/activity of key chaperoned proteins [1, 2, 5–10]. The drugs interacted with sildenafil to inactivate the chaperone GRP78 that was associated with a large increase in PKR-like endoplasmic reticulum (PERK) auto-phosphorylation and with subsequent enhanced down-stream eIF2α phosphorylation [2, 5–10]. The increased expression of the essential autophagosome forming proteins Beclin1 and LC3, caused by the drug combinations, required eIF2α signaling. In addition to GRP78, the drugs interacted with sildenafil to also inactivate the chaperones HSP90 and HSP70 which in turn lost their ability to associate with another chaperone HSP27. Reduced HSP27-HSP90/HSP70 association resulted in altered HSP27 oligomerization and localization without any obviously changes in HSP27 phosphorylation [1, 2]. More importantly, combined loss of GRP78 and HSP27 function reduced signaling through the cyto-protective PI3 kinase pathway, causing a greater level of mTOR inactivation than that of AKT [2]. Reduced mTOR activity resulted in ULK-1 S757 phosphorylation declining and the phosphorylation of the ULK-1 substrate, ATG13 S318, becoming elevated [2, 9]. ATG13 is the gate-keeper for the formation of autophagosomes, with its phosphorylation promoting protein complex formation. Pemetrexed also enhanced ULK-1 S317 phosphorylation, via AMP-dependent kinase signaling, which is also associated with ULK-1 kinase activation [2, 10]. In general agreement with the role of Beclin1, LC3 and ATG13 in the control of autophagosome formation and maturation it was noted that phospho-ATG13 S318 was localized in autophagosomes with Beclin1 and LC3 [2, 10]. Knock down of ATG13 or Beclin1 prevented autophagosome formation and drug combination lethality [2, 10]. Over-expression of GRP78 and HSP27 prevented PERK activation and mTOR inactivation resulting in less autophagosome formation and cell killing [1, 2, 10].

Phosphodiesterase 5 inhibitors (PDE5 inhibitors) are widely used to treat erectile dysfunction [11]. PDE5 is expressed in the wider vasculature and myocardium [11]. Tumor cells can over-express PDE5, as we have demonstrated in hepatoma, breast and NSCLC [7, 9]. PDE5 inhibitors have a well-established safety record and have been shown to be safe in combination with most other medications [12, 13]. The vast majority of studies examining the molecular biology of PDE5 inhibitors have been performed in vascular smooth muscle cells, monocytes and cardiac tissue; not in tumor cells. PDE5 catalyzes the degradation of cyclic GMP (cGMP); i.e. thus PDE5 inhibitors increase cGMP levels [reviewed in 14]. The second messenger nitric oxide (NO) induces smooth muscle relaxation via the actions of cGMP [15–18]. NO at nanomolar levels binds tightly to a heme group in

NO-guanylyl cyclase (GC), also known as soluble guanylyl cyclase, and causes a ~150-fold activation of the enzyme. Activation of NO-GC elevates cGMP levels, which initiate the cGMP signaling pathway, in part through activation of cGMP dependent protein kinase (PKG) [19, 20]. It is known in non-tumor cells that cGMP/PKG, through its stimulatory actions upon the ERK1/2, p38 MAPK, JNK1/2 and NFκB pathways can increase the expression of inducible nitric oxide synthase (iNOS) [21–23]. Thus increased levels of NO activate GC and increase cGMP levels, that activates signaling pathways which increase iNOS levels; and, increased iNOS levels lead to further increases in cellular NO. One mechanism by which NO is inactivated is by its reaction with the superoxide anion (O_2_^−^) [24, 25]. Compared to non-transformed cells, tumor cells generate greater amounts of O_2_^−^. The reaction of NO with O_2_^-^ forms the more potent oxidant peroxynitrite (ONOO^-^) that can damage the functionality of proteins, lipids and DNA [26–30].

The present studies were designed to build upon our work with [pemetrexed + sildenafil], [sorafenib + sildenafil] and [sorafenib + pemetrexed] and were to determine whether PDE5 inhibitors such as sildenafil (Viagra^®^) enhance the anti-tumor effects of [pemetrexed + sorafenib] in non-small cell lung cancer (NSCLC).

## RESULTS

Prior studies have demonstrated using clinically relevant drug concentrations that the NSCLC therapeutic pemetrexed and the liver/renal carcinoma therapeutic sorafenib synergize to killing tumor cells via the induction of toxic autophagosomes [34]. Pemetrexed both causes DNA damage through inhibition of thymidylate synthase and promotes autophagy via ZMP-induced activation of the AMP-dependent protein kinase [31, 32]. Sorafenib, as an inhibitor of chaperones and class III receptor tyrosine kinases, reduces the activity of mTOR and STAT3, and causes endoplasmic reticulum stress signaling that both promotes expression of autophagy regulatory proteins such as Beclin1 and suppresses the expression of short-half-lived protective proteins such as MCL-1. Collectively these double independent actions upon autophagy leads to tumor cell death. The [pemetrexed + sorafenib] drug combination has proved particularly effective in solid tumor patients with a 61% response rate noted [35]. More recently, through PKG-dependent and NO-dependent mechanisms, sildenafil was independently shown to enhance pemetrexed toxicity and sorafenib toxicity [7, 10].

**Figure 1:**
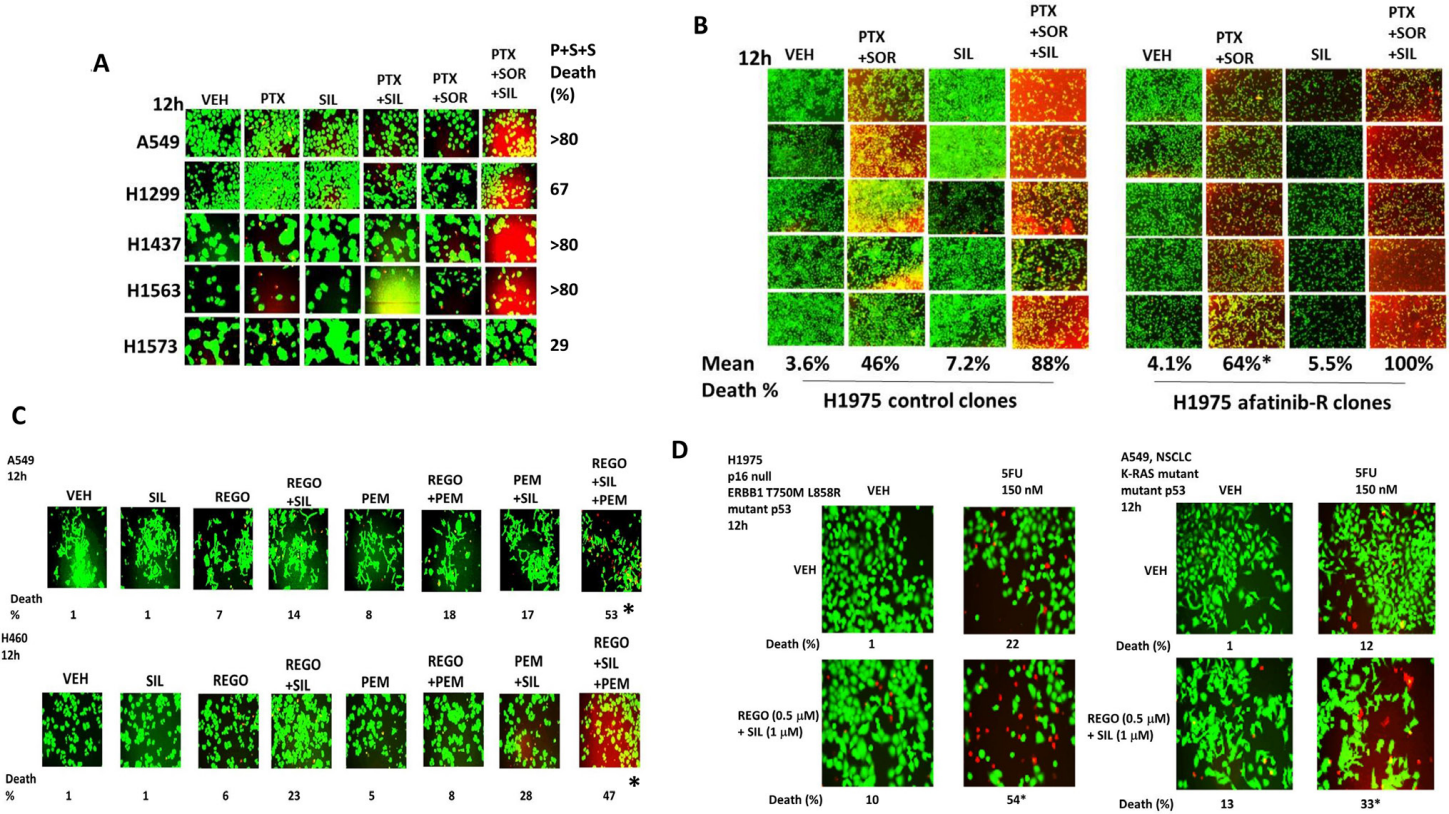
Sildenafil enhances the lethality of [pemetrexed + sorafenib] (**A**) NSCLC cells were treated for 12 h with vehicle control, pemetrexed (1.0 μM), sildenafil (2.0 μM), sorafenib (2.0 μM) or the drugs in combination as indicated. Floating cells were then cytospun onto the 96 well plate and cell viability determined using a live/dead viability stain where green cells are viable and yellow / red cells are dead in WiScan Hermes instrument. The percentage cell death in cells treated with [pemetrexed + sorafenib + sildenafil] is shown; all are statistically significantly greater than the killing caused by [pemetrexed + sildenafil] or [pemetrexed + sorafenib] (*p* < 0.05). (**B**) Parental clones of H1975 cells and afatinib resistant clones of H1975 cells were treated for 12 h with vehicle control, pemetrexed (1.0 μM), sildenafil (2.0 μM), sorafenib (2.0 μM) or the drugs in combination as indicated. Floating cells were then cytospun onto the 96 well plate and cell viability determined. The percentage cell death in afatinib resistant cells treated with [pemetrexed + sorafenib] is statistically significantly greater than the killing caused by [pemetrexed + sorafenib] in parental cells (*p < 0.05). (**C**) NSCLC cells were treated for 12 h with vehicle control, pemetrexed (1.0 μM), sildenafil (2.0 μM), regorafenib (0.5 μM) or the drugs in combination as indicated. Floating cells were then cytospun onto the 96 well plate and cell viability determined. The percentage cell death in cells treated with [pemetrexed + regorafenib + sildenafil] is shown; all data are statistically significantly greater than the killing caused by [pemetrexed + sildenafil] or [pemetrexed + regorafenib] (*p < 0.05). (**D**) NSCLC cells were treated for 12 h with vehicle control, 5-fluoro-uracil (5FU) (150 nM), sildenafil (2.0 μM), regorafenib (0.5 μM) or the drugs in combination as indicated. Floating cells were then cytospun onto the 96 well plate and cell viability determined. The percentage cell death in cells treated with [5FU + regorafenib + sildenafil] is shown; all data are statistically significantly greater than the killing caused by [regorafenib + sildenafil] or 5FU (*p < 0.05).

Thus, our initial studies determined in a genetically diverse set of NSCLC cells whether the phosphodiesterase 5 inhibitor sildenafil interacted with the combination of the lung and liver cancer therapeutics [pemetrexed + sorafenib], to enhance tumor cell killing. *In vitro* drug concentrations in the present manuscript were chosen based on the reported C max values of the drugs in patients; cells are treated with drugs in the 1% (pemetrexed) – 20% (sorafenib) - 100% (sildenafil) range of that safely found in patient plasma. To varying degrees, sildenafil enhanced the killing potential of [pemetrexed + sorafenib] in lung cancer cells (Figure 1A). The three drug combination was equally effective at killing in wild type and *in vivo* generated afatinib resistant H1975 cells (Figure 1B). The colon cancer therapeutic regorafenib as a single agent was less effective than sorafenib at enhancing pemetrexed lethality, whereas pemetrexed combined with both regorafenib and sildenafil caused high levels of tumor cell death (Figure 1C). The older thymidylate synthase inhibitor drug 5-fluorouracil (5FU), that unlike pemetrexed has not proposed to elevate ZMP levels, also combined with regorafenib and sildenafil to kill NSCLC cells (Figure 1D).

**Figure 2:**
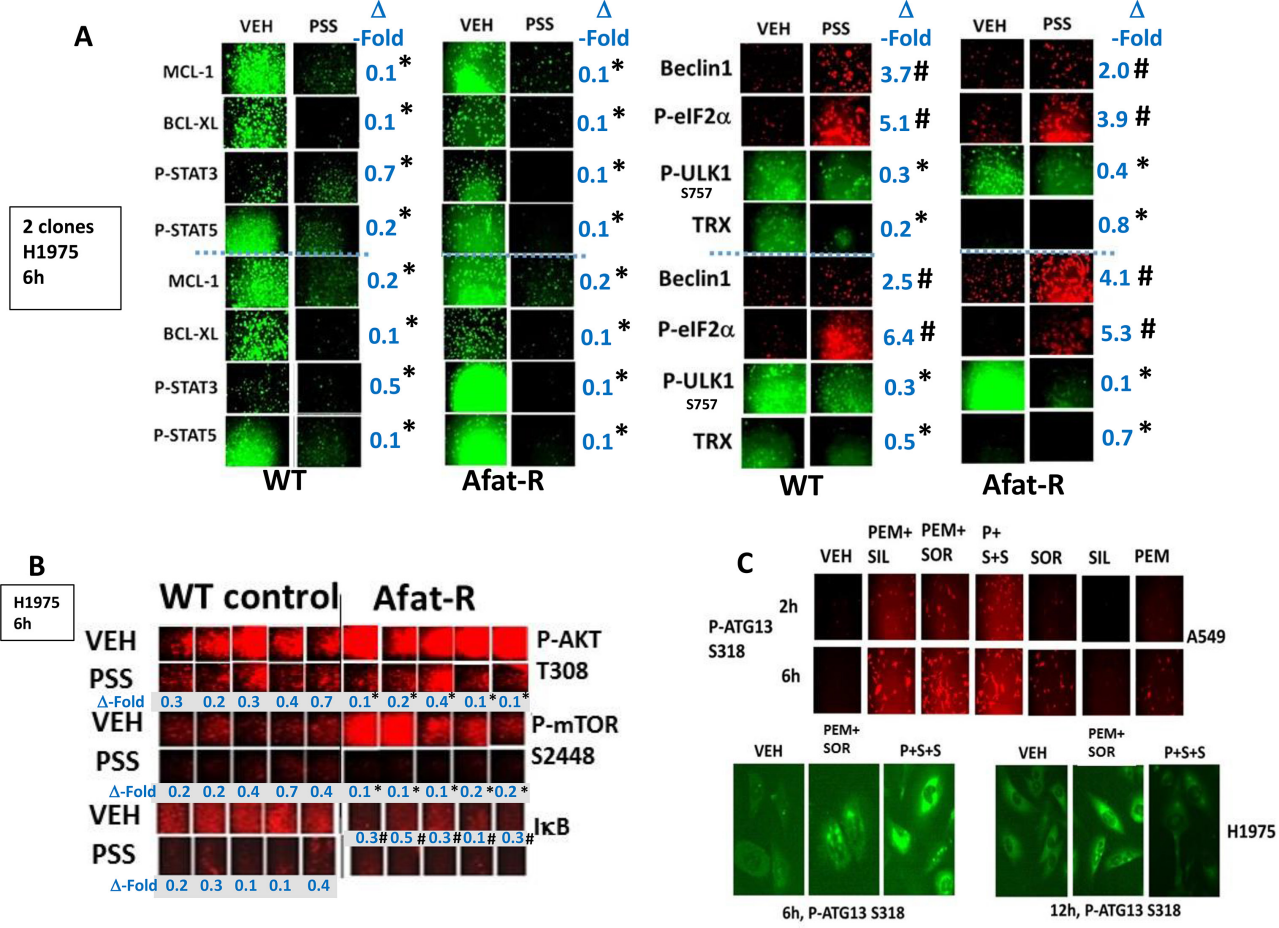
[Pemetrexed + sorafenib + sildenafil] treatment inactivates cyto-protective STAT3, STAT5 and AKT whilst reducing the expression of cyto-protective proteins MCL-1, BCL-XL and Thioredoxin (**A**) Parental clones of H1975 cells and afatinib resistant clones of H1975 cells were treated for 6 h with vehicle control or with [pemetrexed (1.0 μM), sildenafil (2.0 μM), sorafenib (2.0 μM)] in combination as indicated. Cells were fixed in place and immuno-fluorescence staining performed to determine the phosphorylation and expression of the indicated proteins *p < 0.05 less than corresponding intensity in vehicle control cells; ^#^*p* < 0.05 greater than corresponding intensity in vehicle control cells. (**B**) Parental clones of H1975 cells and afatinib resistant clones of H1975 cells were treated for 6 h with vehicle control or with [pemetrexed (1.0 μM), sildenafil (2.0 μM), sorafenib (2.0 μM)] in combination as indicated. Cells were fixed in place and immuno-fluorescence staining performed to determine the phosphorylation and expression of the indicated proteins. *p < 0.05 less than the mean of the corresponding values in wild type control cells; ^#^*p* < 0.05 less than the mean of the corresponding vehicle treated values in wild type control cells. (**C**) A549 and H1975 NSCLC cells were treated for 2 h–12 h as indicated with vehicle control or with pemetrexed (1.0 μM), sildenafil (2.0 μM), sorafenib (2.0 μM), alone or in combination as indicated. Cells were fixed in place and immuno-fluorescence staining performed to determine the phosphorylation and expression of the indicated proteins.

**Figure 3:**
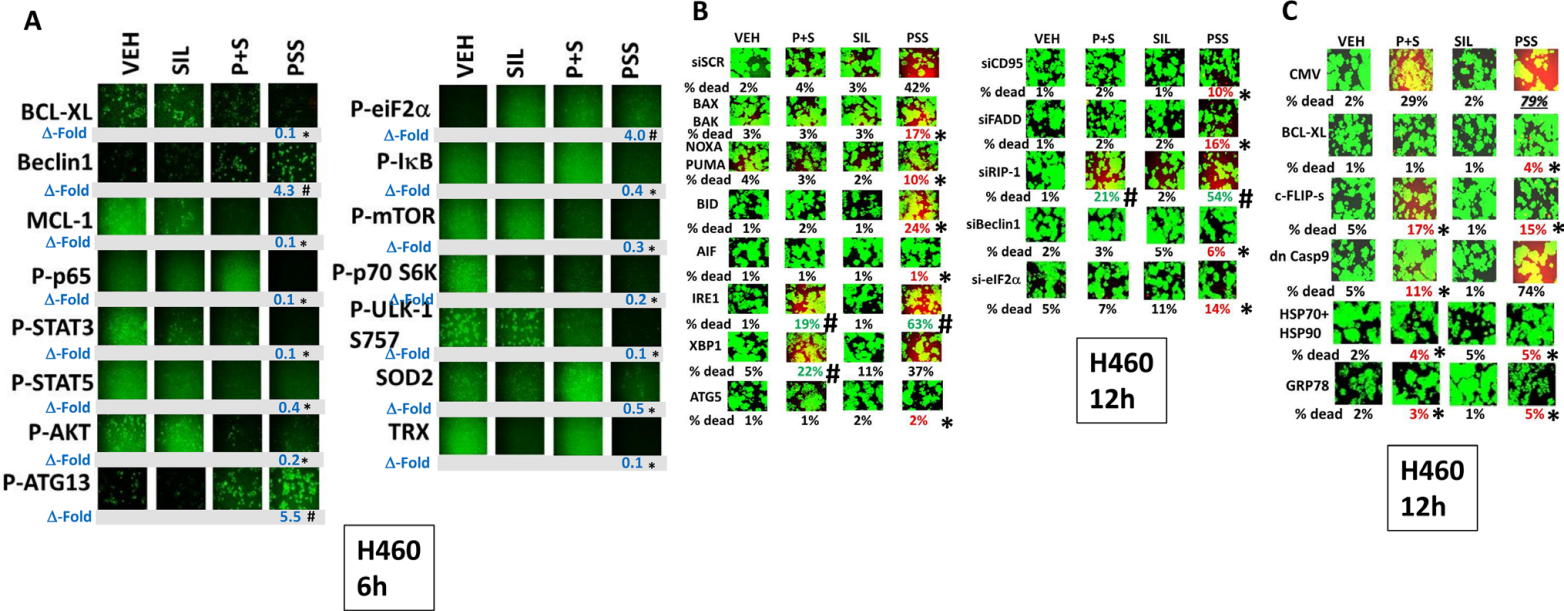
Treatment of cells with [pemetrexed + sorafenib + sildenafil] more effectively inactivates NFκB, mTOR and STAT transcription factors than [pemetrexed + sorafenib] (**A**) H460 cells were treated for 6 h with vehicle control or with pemetrexed (1.0 μM), sildenafil (2.0 μM), sorafenib (2.0 μM), alone or in combination as indicated. Cells were fixed in place and immuno-fluorescence staining performed to determine the phosphorylation and expression of the indicated proteins. *p < 0.05 less than corresponding intensity in vehicle control cells; ^#^*p* < 0.05 greater than corresponding intensity in vehicle control cells. (**B**) H460 cells were transfected with a scrambled siRNA (siSCR) or with molecules to knock down the expression of: BAX + BAK; NOXA + PUMA; BID; AIF; IRE1; XBP1; ATG5; CD95; FADD; RIP-1; Beclin1; or eIF2α. Twenty-four h after transfection cells were treated for 12 h with vehicle control or with pemetrexed (1.0 μM), sildenafil (2.0 μM), sorafenib (2.0 μM), alone or in combination as indicated. Floating cells were then cytospun onto the 96 well plate and cell viability determined. The percentage cell death under each transfection and treatment condition was calculated and values with a statistical significance lower than the corresponding value CMV cells are in red; those whose value is greater than in CMV cells are green (*p < 0.05 less than corresponding value in siSCR cells; ^#^*p* < 0.05 greater than corresponding value in siSCR cells). (**C**) H460 cells were transfected with an empty vector plasmid (CMV) or with plasmids to express: BCL-XL; c-FLIP-s; dominant negative caspase 9; HSP90 + HSP70; or GRP78. Twenty-four h after transfection cells were treated for 12 h with vehicle control or with pemetrexed (1.0 μM), sildenafil (2.0 μM), sorafenib (2.0 μM), alone or in combination as indicated. Floating cells were then cytospun onto the 96 well plate and cell viability determined. The percentage cell death under each transfection and treatment condition was calculated and values with a statistical significance lower than the corresponding value CMV cells are in red; those whose value is greater than in CMV cells are green (*p < 0.05 less than corresponding value in CMV cells).

Afatinib-resistant H1975 lung cancer cells were generated as part of the project that demonstrated ERBB1/2/4 inhibitors enhanced [pemetrexed + sildenafil] killing [2]. The resistant H1975 cells did not contain any additional hot spot mutations when compared to wild type cells but exhibited high levels of SRC-dependent ERBB3 phosphorylation and increased expression of c-MET and c-KIT [2, 37]. Treatment of wild type and afatinib resistant H1975 cells with [pemetrexed + sorafenib + sildenafil] reduced the expression of the mitochondrial protective proteins MCL-1 and BCL-XL and the reactive oxygen species detoxifying protein thioredoxin (TRX) (Figure 2A). The phosphorylation of ULK-1 S757, STAT3, STAT5, mTOR and AKT was reduced and the phosphorylation of eIF2α enhanced (Figure 2A and 2B). Six hours after drug combination exposure, in agreement with ULK-1 S757 dephosphorylation, the phosphorylation of ATG13 S318 was elevated, prior to any observed actual cell killing; in cells treated with the three drug combination the levels of phospho-ATG13 S318 were marginally higher than those in cells only treated with pemetrexed and sorafenib (Figure 2C). Of greater note was that 12 h after drug exposure, at a time when three drug treated cells were undergoing cell death, the levels of phospho-ATG13 S318 had declined. In A549 and H460 cells three-drug treatment, as well as two-drug pemetrexed and sorafenib treatment of lung cancer cells also increased the phosphorylation of eIF2α S51, indicative of endoplasmic reticulum stress, and ATG13 S318 whereas it decreased the phosphorylation of AKT T308, p70 S6K T389, mTOR S2448 and ULK-1 S757 (Figure 3 and Supplementary Figure 1). These drug-induced changes in phosphorylation were associated with reduced expression of the protective BCL-XL and MCL-1 proteins, and increased expression of the autophagy regulatory protein Beclin1.

As eIF2α was phosphorylated after drug combination exposure, we next determined the relative importance of the known endoplasmic reticulum stress signaling pathways in the death and/or survival of tumor cells treated with [pemetrexed + sorafenib]. In H460 cells over-expression of BCL-XL, the caspase 8/10 inhibitor c-FLIP-s, [HSP70 + HSP90] or GRP78 significantly reduced the lethality of [pemetrexed + sorafenib + sildenafil] treatment (Figure 3C). Knock down of the death receptor CD95, the death receptor docking protein FADD, Beclin1, ATG5, the necroptotic DNA digesting enzyme AIF or eIF2α reduced the lethality of [pemetrexed + sildenafil] and [pemetrexed + sorafenib + sildenafil] whereas knock down of XBP-1, part of the IRE1 endoplasmic reticulum stress signaling pathway, enhanced killing. Very similar cell viability data were obtained in H460 and A549 NSCLC cells (Supplementary Figures 3, 4 and 5).

**Figure 4:**
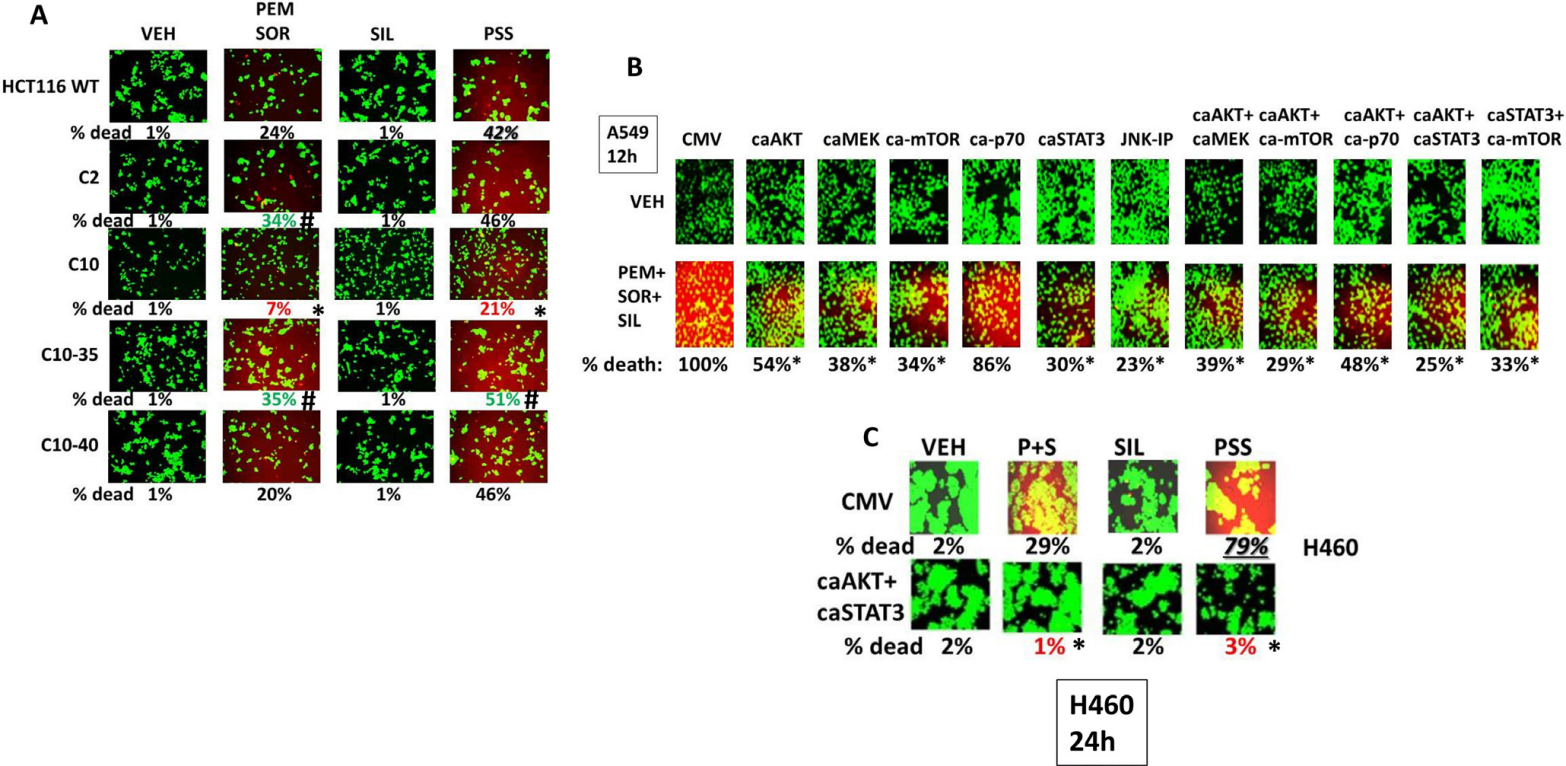
Differential regulation of resistance to [pemetrexed + sorafenib + sildenafil] by the PI3K, ERK1/2 and STAT signaling pathways (**A**) HCT116 cells (parental wild type; K-RAS D13 deleted, C2; C2 cells transfected to express H-RAS V12, C10; C2 cells transfected to express H-RAS V12 C10-35 that activates the ERK1/2 pathway; C2 cells transfected to express H-RAS V12 C10-40 that activates the PI3K pathway) were treated for 12 h with vehicle control or with pemetrexed (1.0 μM), sildenafil (2.0 μM), sorafenib (2.0 μM), alone or in combination as indicated. Floating cells were then cytospun onto the 96 well plate and cell viability determined. The percentage cell death under each transfection and treatment condition was calculated and values with a statistical significance lower than the corresponding value CMV cells are in red; those whose value is greater than in CMV cells are green (*p < 0.05 less than corresponding value in wild type cells; ^#^*p* < 0.05 greater than corresponding value in wild type cells). (**B**) A549 cells were transfected with an empty vector plasmid (CMV) or with plasmids to express: activated AKT; activated MEK1; activated mTOR; activated p70 S6K; alone or in the indicated combinations. CMV transfected cells were treated with the JNK inhibitory peptide as indicated (10 μM). Twenty-four h after transfection were treated for 12 h with vehicle control or with pemetrexed (1.0 μM), sildenafil (2.0 μM), sorafenib (2.0 μM), alone or in combination as indicated. Floating cells were then cytospun onto the 96 well plate and cell viability determined. The percentage cell death under each transfection and treatment condition was calculated and values with a statistical significance lower than the corresponding value CMV cells (*p < 0.05 less than corresponding value in CMV cells). (**C**) H460 cells were transfected with an empty vector plasmid (CMV) or with plasmids to express: activated AKT and activated STAT3. Twenty-four h after transfection were treated for 12 h with vehicle control or with pemetrexed (1.0 μM), sildenafil (2.0 μM), sorafenib (2.0 μM), alone or in combination as indicated. Floating cells were then cytospun onto the 96 well plate and cell viability determined. The percentage cell death under each transfection and treatment condition was calculated and values with a statistical significance lower than the corresponding value CMV cells are in red; those whose value is greater than in CMV cells are green (*p < 0.05 less than corresponding value in CMV cells).

**Figure 5:**
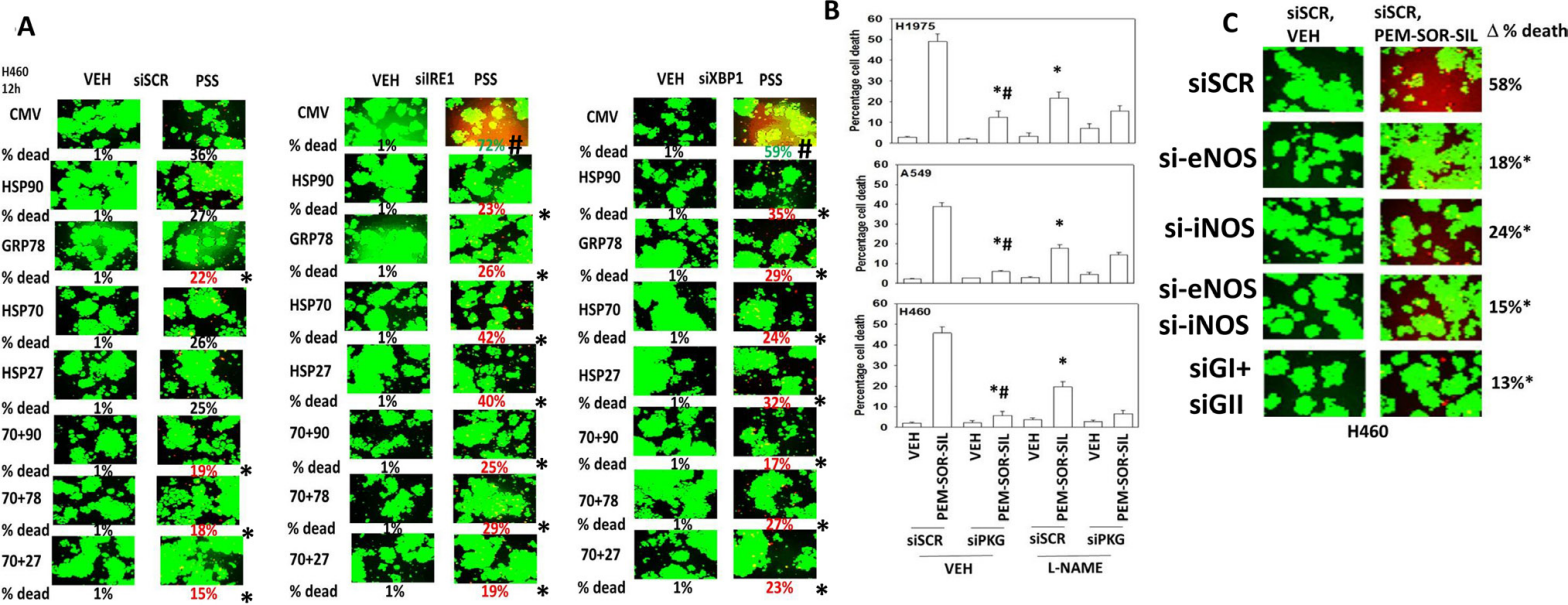
Sildenafil-induced PKG signaling plays a greater role in enhancing [pemetrexed + sorafenib] toxicity than nitric oxide synthase signaling (**A**) H460 cells were transfected with a scrambled siRNA (siSCR) or molecules to knock down the expression of IRE1 or XBP1. In parallel, cells were transfected with an empty vector plasmid (CMV) or with plasmids to express: HSP90; GRP78; HSP70; HSP27 or in the indicated combinations. Twenty-four h after transfection cells were treated with vehicle control or [pemetrexed (1.0 μM) + sildenafil (2.0 μM) + sorafenib (2.0 μM)] for 12 h. Floating cells were then cytospun onto the 96 well plate and cell viability determined. The percentage cell death under each transfection and treatment condition was calculated and values with a statistical significance lower than the corresponding value CMV cells are in red; those whose value is greater than in CMV cells are green (*p < 0.05 less than corresponding CMV cells; ^#^*p* < 0.05 greater than corresponding value in siSCR cells). (**B**) NSCLC cells were transfected with a scrambled siRNA control (siSCR) or transfected to knock down the expression of PKGI and PKGII. Twenty-four h after transfection cells were treated with vehicle control or [pemetrexed (1.0 μM) + sorafenib (2.0 μM) + sildenafil (2.0 μM)] in combination for 24 h. Thirty minutes prior to drug treatment, cells were treated with vehicle control or with the nitric oxide synthase inhibitor L-NAME (1 μM). Floating cells were cytospun onto the 96 well plate and viability determined using a live/dead viability stain where green cells are viable and yellow/red cells are dead (*n* = 3 +/−SEM) *p < 0.05 less than corresponding value in siSCR cells; ^#^*p* < 0.05 less than corresponding value in siSCR + L-NAME cells. (**C**) NSCLC cells were transfected with a scrambled control siRNA (siSCR) or with siRNA molecules to knock down the expression of: iNOS and eNOS; PKGI and PKGII, as indicated. Twenty-four h after transfection cells were treated with vehicle control or [pemetrexed (1.0 μM) + sorafenib (2.0 μM) + sildenafil (2 μM)] in combination for 24 h. Floating cells were cytospun onto the 96 well plate and viability determined using a live/dead viability stain where green cells are viable and yellow/red cells are dead (*n* = 3 +/−SEM) *p < 0.05 less than corresponding value in siSCR cells; ^#^*p* < 0.05 less than values in individual NOS knock down cells.

Prior studies had created a series of HCT116 colon cancer cell clones that expressed K-RAD D13 (wild type parental) as well as HCT116 clones that were deleted for K-RAS D13 and instead expressed H-RAS V12; H-RAS V12-35 that specifically activates ERK1/2; H-RAS V12 that specifically activates PI3K [38, 39]. Deletion of K-RAS D13, termed C2 cells, significantly enhanced the lethality of [pemetrexed + sorafenib] but not of the three drug combination (Figure 4A). In contrast, expression of H-RAS V12 significantly reduced the lethality of both the two and three drug combinations. Expression of H-RAS V12-35 did not protect cells from either of the drug combinations with killing similar to that in the C2 cells that lack expression of any mutated active RAS protein. Expression of H-RAS V12-40 maintained cell sensitivity to the two drug combinations at a level comparable to that observed in the wild type HCT116 cells expressing K-RAS D13. In A549 cells that express a mutated active K-RAS expression of activated forms of AKT, MEK1, mTOR or STAT3 variably reduced drug-induced killing by 50–70%, as did inhibition of the JNK pathway (Figure 4B). Similar data were observed in H460 cells over-expressing activated AKT and STAT3 (Figure 4C). In wild type and afatinib H1975 cells that express a double mutated active ERBB1 expression of activated forms of AKT, MEK1, mTOR, p70 S6K or STAT3 reduced drug-induced killing by > 75% (Supplementary Figure 6). Knock down of the IRE1-XBP-1 pathway enhanced killing by [pemetrexed + sorafenib + sildenafil] (Figure 5A). Over-expression of multiple chaperone proteins could circumvent the enhanced tumor cell killing by IRE1-XBP-1 pathway knock down (Figure 5A and Supplementary Figure 7).

The roles of cGMP and nitric oxide in the regulation of pemetrexed toxicity were next investigated. Knock down of PKGI and PKGII expression together significantly reduced the ability of sildenafil to enhance [pemetrexed + sorafenib] toxicity (Figure 5B). Pan-inhibition of nitric oxide synthase (NOS) enzymes using the chemical compound L-NAME also significantly reduced the ability of sildenafil to enhance pemetrexed lethality although this effect was significantly less than that afforded by knock down of PKGI/II. We then determined which NOS enzymes were responsible for facilitating the sildenafil effect. We did not observe nNOS expression in our cells (data not shown). In H1975 cells knock down of eNOS and iNOS, but not either individually, reduced [pemetrexed + sildenafil] lethality (Figure 5C and Supplementary Figure 8). In H460 and A549 cells knock down of either iNOS or eNOS reduced drug combination lethality (Figure 5C and Supplementary Figure 8).

**Figure 6:**
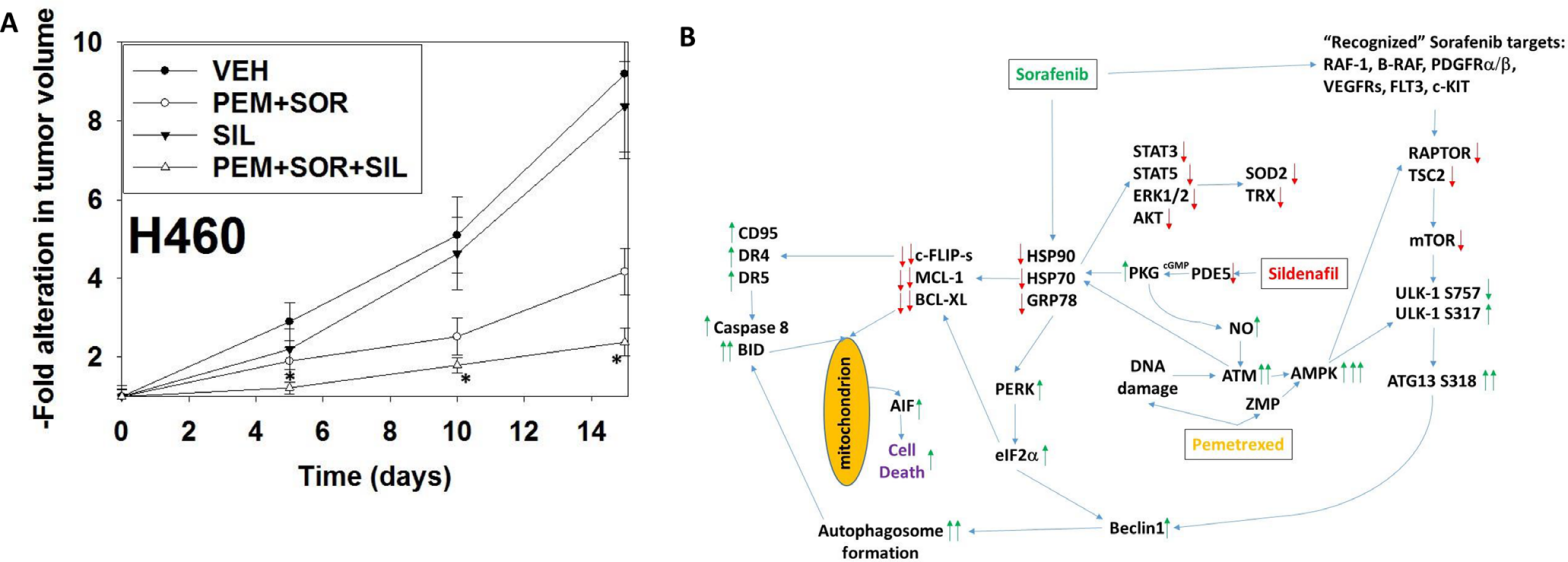
Sildenafil enhances the anti-tumor effects of pemetrexed *in vivo* (**A**) H460 cells into the rear flank of male athymic mice (the H460 cell line was isolated from a male patient). Tumors were permitted to form and the animals treated with vehicle control, pemetrexed, sorafenib and sildenafil as described in the Methods section and as indicated in the graphical panel. *p < 0.05 less than [pemetrexed + sorafenib] treatment. (**B**) The mechanisms by which sildenafil enhances the lethality of [pemetrexed + sorafenib]. See also reference 10, Figure 14. As a thymidylate synthase inhibitor pemetrexed causes DNA damage and increases the levels of ZMP due to inhibition of AICAR. Sildenafil, as a PDE5 inhibitor, increases the levels of cGMP which activates PKG and subsequently leads to the generation of nitric oxide. Nitric oxide enhances the activation of ATM caused initially by DNA damage. ATM signals to activate the AMPK which is further allosterically activated by ZMP. Collectively this strong AMPK signal inactivates RAPTOR and TSC2 resulting in the inactivation of mTORC1 and mTORC2. Downstream of mTOR is the kinase ULK-1; the drug combination via AMPK promotes ULK-1 S317 phosphorylation which activates the kinase; the drug combination via mTOR inactivation reduces ULK-1 S757 phosphorylation which also activates the kinase. Activated ULK-1 phosphorylates ATG13 which is the key gate-keeper step in permitting autophagosome formation. Sildenafil-induced PKG signaling also acts to reduce the activities of multiple chaperone proteins which is augmented by DNA damage induced ATM signaling. Sorafenib, in addition to inhibiting class III receptor tyrosine kinases, RAF-1 and B-RAF, also reduces GRP78, HSP90 and HSP70 function that in turn lowers the activities of STAT3, STAT5, ERK1/2 and AKT that results in lower expression of ROS/RNS detoxifying enzymes such as TRX and SOD2. Reduced GRP78 function causes activation of PERK and subsequently eIF2α. Enhanced eIF2α signaling reduces the transcription of proteins with short half-lives such as c-FLIP-s, MCL-1 and BCL-XL, and enhances expression of Beclin1, DR4 and DR5. Thus the convergent actions of reduced GRP78, HSP90 and HSP70 chaperone activity and eIF2α signaling lead to a profound reduction in the protein levels of c-FLIP-s, MCL-1 and BCL-XL which facilitates death receptor signaling through CD95, DR4 and DR5 to activate the extrinsic apoptosis pathway. ER stress-induced elevations of Beclin1 expression converge with elevated ATG13 phosphorylation to produce high levels of autophagosome formation that, when fused with lysosomes and releasing proteases into the cytosol, converges with the extrinsic apoptosis pathway to cleave BID and cause mitochondrial dysfunction. Tumor cell killing downstream of the mitochondrion was mediated by AIF and not caspases 3/7. The tumoricidal actions of AIF were facilitated by sorafenib reducing HSP70 functionality as this chaperone can sequester AIF in the cytosol and prevent its translocation to the nucleus.

Finally, we determined whether sildenafil interacted with [pemetrexed + sorafenib] *in vivo* to suppress lung tumor growth (Figure 6A). Treatment with [pemetrexed + sorafenib], but not sildenafil, reduced the growth of H460 NSCLC tumors. However, the reduction in tumor growth in animals treated with [pemetrexed + sildenafil + sorafenib] was significantly greater than either individual treatment. By Day 15, 12 days after the cessation of therapy vehicle control treated tumors had increased their volume 8.3-fold whereas those treated with [pemetrexed + sildenafil + sorafenib] had increased their volume 2.4-fold.

## DISCUSSION

In the companion manuscript to the present studies we demonstrated that PDE5 inhibitors such as sildenafil (Viagra^®^) and tadalafil (Cialis^®^) could enhance the lethality of the NSCLC standard-of-care medication pemetrexed (Alimta^®^) [10]. The studies in this manuscript attempted to build upon these findings and determine whether PDE5 inhibitors could enhance the killing potential of the clinically relevant drug combination of [pemetrexed + sorafenib] (see also NCT01450384; NCT02624700; NCT02466802). In a manner similar to our prior studies combining [sorafenib/regorafenib + sildenafil] and [pemetrexed + sorafenib + afatinib] we found that the ability of sildenafil to increase the levels of both cGMP and nitric oxide played key roles in facilitating a more robust increase in the numbers of toxic autophagosomes and tumor cell killing. Based on our prior studies in multiple tumor cell types using sildenafil as an activity enhancer of established chemotherapies we suggest that this agent may have a broad utility in future chemotherapeutic approaches.

In this manuscript we also extended our analyses from using sorafenib to its cousin, the more soluble fluorinated and potent drug regorafenib. In comparison to our prior studies combining [pemetrexed + sorafenib], the combination of [pemetrexed + regorafenib] was much less effective at killing tumor cells. We know that as a single agent, when compared to sorafenib, regorafenib is a much weaker inhibitor of the HSP90 and HSP70 chaperone ATPase activities [1]. Regorafenib as a single agent, also, is less capable of causing an NH2-terminal conformational change in chaperone tertiary structure when compared to sorafenib, however, when combined with sildenafil regorafenib becomes competent to inhibit chaperone ATPase activities and to cause the NH2-terminal conformational change [1, 5]. In our present studies we found that [regorafenib + sildenafil] interacted with both pemetrexed and the first generation thymidylate synthase inhibitor 5-fluorouracil to kill in a greater than additive fashion. As a phase I trial combining [pemetrexed + sorafenib] with positive results has been completed (NCT01450384), and as a phase I trial combining [regorafenib + sildenafil] is nearing completion (NCT02466802), our present *in vitro* and animal studies herein suggest that the combination of [regorafenib + sildenafil + 5FU/pemetrexed] should be considered as a phase I trial in all solid tumor patients [35].

The combination of [pemetrexed + sorafenib + sildenafil] fully inactivated signaling by multiple cyto-protective proteins including the AKT/ERK pathways, NFκB and STAT3/STAT5 (Figure 6B). These changes in activity were associated with reduced protein levels of the mitochondrial/ER protective proteins BCL-XL and MCL-1, and of the reactive oxygen species detoxifying enzymes thioredoxin and super-oxide dismutase. In the companion manuscript we found that one major/essential component of the killing process by [pemetrexed + sildenafil] was through increased death receptor expression/death receptor activation (Figure 6B) [10]. Our data with the three drug combination is however more nuanced, with sildenafil in some cell types enhancing [pemetrexed + sorafenib] lethality through death receptor signaling whereas in other cell types the three drug combination does not appear to heavily rely on signaling by death receptors. In some of the tumor cell types tested, the restoration of signaling through multiple cyto-protective pathways, e.g. AKT and STAT3 in H460 cells, was required to significantly suppress killing by the three drug combination whereas in H1975 cells expression of activated AKT or of activated STAT3 exhibited strong cyto-protective effects. In HCT116 colon cancer cells that we had previously genetically manipulated to express various mutant active H-RAS proteins we discovered that loss of mutant active K-RAS D13 signaling enhanced the lethality of [pemetrexed + sorafenib] but did not further enhance killing by the three drug combination. Expression of H-RAS V12 that could activate both the PI3K and ERK1/2 pathways strong suppressed all drug-induced killing processes and an H-RAS V12 protein that could only activate PI3K showed similar protective effects to those afforded by K-RAS D13. Expression of an H-RAS V12 mutant that could only activate the ERK1/2 pathway actually increased the lethality of the drug combinations which is congruent with our prior work which demonstrated that expression of a dominant negative MEK1 protein suppressed [pemetrexed + sorafenib] killing.

A number of our prior studies using sorafenib and sildenafil had linked the biological action of these agents in combination to the inhibition of HSP90 family and HSP70 family chaperones [1, 2, 5, 8, 10]. As was noted in the companion manuscript we observed the over-expression of chaperones, particularly when expressed in combination reduced the killing potential of [pemetrexed + sorafenib + sildenafil] [10]. Also, as was observed in the companion study, we demonstrated that PKG-dependent signaling downstream of sildenafil played a greater role in enhancing the anti-tumor effect of [pemetrexed + sorafenib] than did generation of nitric oxide.

In an animal model of NSCLC, we found that sildenafil interacted in a greater than additive fashion to suppress the growth of H460 tumors. In the companion paper [pemetrexed + sildenafil] treatment reduced the -fold increase in H460 tumor volume from 8.3-fold in vehicle treated animals to 3.2-fold for drug treated tumors [10]. For tumors treated with [pemetrexed + sorafenib + sildenafil] the -fold increase in volume was only 2.4-fold, that was significantly lower than the anti-tumor effect of [pemetrexed + sildenafil] (*p* < 0.05). At present a phase II trial combining pemetrexed and sorafenib in previously treated triple negative breast cancer patients is open at VCU Massey Cancer Center, with the first patient a confirmed partial response (NCT02624700 (Poklepovic and Dent, unpublished observations). It will be of interest, should this trial confirm the efficacy of [pemetrexed + sorafenib] in this patient population, whether a new trial can be initiated adding sildenafil to the drug combination.

## MATERIALS AND METHODS

### Materials

Pemetrexed was purchased from LC Laboratories (Woburn, MA). Sildenafil and sorafenib tosylate was purchased from Selleckchem (Houston, TX). Trypsin-EDTA, DMEM, RPMI, penicillin-streptomycin were purchased from GIBCOBRL (GIBCOBRL Life Technologies, Grand Island, NY). Cells were purchased from the ATCC and were not further validated beyond that claimed by ATCC. Cells were re-purchased every ~6 months. ADOR is a primary NSCLC isolate donated to the Dent laboratory by the patient. The plasmid to express GRP78/BiP/HSPA5 was kindly provided to the Dent laboratory by Dr. A.S. Lee (University of Southern California, Los Angeles, CA); all other plasmids were purchased from Addgene. Commercially available validated short hairpin RNA molecules to knock down RNA/protein levels were from Qiagen (Valencia, CA) (Supplementary Figure 8). Reagents and performance of experimental procedures were described in refs: [1, 2, 6–10].

### Methods

#### Culture and *in vitro* exposure of cells to drugs

All cell lines were cultured at 37^°^C (5% (v/v CO_2_) *in vitro* using RPMI supplemented with dialyzed 5% (v/v) fetal calf serum and 10% (v/v) Non-essential amino acids. Cells growing in “complete” fetal calf serum that contains thymidine were gradually weaned into dialyzed serum lacking thymidine over 2 weeks and were then used for experimental analyses for the following 3 weeks before discarding. Cells were re-isolated in thymidine-less media as required. For short term cell killing assays, immunoblotting studies, cells were plated at a density of 3 × 10^3^ per cm^2^ (~2 × 10^5^ cells per well of a 12 well plate) and 48 h after plating treated with various drugs, as indicated. *In vitro* pemetrexed, sorafenib, regorafenib, sildenafil and other drug treatments were generally from a 100 mM stock solution of each drug and the maximal concentration of Vehicle carrier (VEH; DMSO) in media was 0.02% (v/v). Cells were not cultured in reduced serum media during any study in this manuscript.

### Transfection of cells with siRNA or with plasmids

#### For Plasmids

Cells were plated and 24 h after plating, transfected. Plasmids expressing a specific mRNA (or siRNA) or appropriate vector control plasmid DNA was diluted in 50 μl serum-free and antibiotic-free medium (1 portion for each sample). Concurrently, 2 μl Lipofectamine 2000 (Invitrogen), was diluted into 50 μl of serum-free and antibiotic-free medium (1 portion for each sample). Diluted DNA was added to the diluted Lipofectamine 2000 for each sample and incubated at room temperature for 30 min. This mixture was added to each well/dish of cells containing 200 μl serum-free and antibiotic-free medium for a total volume of 300 μl, and the cells were incubated for 4 h at 37°C. An equal volume of 2× medium was then added to each well. Cells were incubated for 24 h, then treated with drugs.

### Transfection for siRNA

Cells from a fresh culture growing in log phase as described above, and 24 h after plating transfected. Prior to transfection, the medium was aspirated and serum-free medium was added to each plate. For transfection, 10 nM of the annealed siRNA, the positive sense control doubled stranded siRNA targeting GAPDH or the negative control (a “scrambled” sequence with no significant homology to any known gene sequences from mouse, rat or human cell lines) were used. Ten nM siRNA (scrambled or experimental) was diluted in serum-free media. Four μl Hiperfect (Qiagen) was added to this mixture and the solution was mixed by pipetting up and down several times. This solution was incubated at room temp for 10 min, then added drop-wise to each dish. The medium in each dish was swirled gently to mix, then incubated at 37^°^C for 2 h. Serum-containing medium was added to each plate, and cells were incubated at 37^°^C for 24 h before then treated with drugs (0–24 h). Additional immuno-fluorescence/live-dead analyses were performed at the indicated time points.

### Animal studies

Studies were performed according to USDA regulations under VCU IACUC protocol AD20008. Athymic nude mice (~20 g) were injected with 1 × 10^7^ H460 cells into their rear flank (10 animals per treatment group; 4 groups; a total of 40 mice +/− SEM). Tumors were permitted to form for 7 days with tumors at that time exhibiting a mean volume of ~25 mm^3^. Athymic mice were treated by oral gavage once every day QD for four days as indicated in the Figure and Figure Legend with vehicle (Cremophore); with pemetrexed (50 mg/kg) only on day 1; with sildenafil (5 mg/kg) on days 1–3 and/or with sorafenib (25 mg/kg) on Days 1–3. After cessation of drug treatment tumors are again calipered as indicated in the Figure and tumor volume was assessed up to 20–30 days later.

Detection of cell viability, protein expression and protein phosphorylation by immuno-fluorescence using a Hermes WiScan machine. http://www.idea-bio.com/ Cells (4 × 10^3^) are plated into each well of a 96 well plate, and cells permitted to attach and grow for the next 18 h. Based on the experiment, after 18 h, cells are then either genetically manipulated, or are treated with drugs. For genetic manipulation, cells are transfected with plasmids or siRNA molecules and incubated for an additional 24 h. Cells are treated with vehicle control or with drugs at the indicated final concentrations, alone or in combination. Cells are then isolated for processing at various times following drug exposure. The 96 well plate is centrifuged/cyto-spun to associate dead cells (for live-dead assays) with the base of each well. For live dead assays, after centrifugation, the media is removed and cells treated with live-dead reagent (Thermo Fisher Scientific, Waltham MA) and after 10 min this is removed and the cells in each well are visualized in the Hermes instrument at 10X magnification. Green cells = viable; yellow/red cells = dying/dead. The numbers of viable and dead cells were counted manually from three images taken from each well combined with data from another two wells of separately treated cells (i.e. the data is the mean cell dead from 9 data points from three separate exposures). For immuno-fluorescence studies, after centrifugation, the media is removed and cells are fixed in place and permeabilized using ice cold PBS containing 0.4% paraformaldehyde and 0.5% Triton X-100. After 30 min the cells are washed three times with ice cold PBS and cells are pre-blocked with rat serum for 3 h. Cells are then incubated with a primary antibody to detect the expression/phosphorylation of a protein (usually at 1:100 dilution from a commercial vendor) overnight at 37°C. Cells are washed three times with PBS followed by application of the secondary antibody containing an associated fluorescent red or green chemical tag. After 3 h of incubation the antibody is removed and the cells washed again. The cells are visualized at either 10× or 60× in the Hermes machine for imaging assessments. All immunofluorescent images for each individual protein/phospho-protein are taken using the identical machine settings so that the levels of signal in each image can be directly compared to the level of signal in the cells treated with drugs. Similarly, for presentation, the enhancement of image brightness/contrast using PhotoShop CS6 is simultaneously performed for each individual set of protein/phospho-protein to permit direct comparison of the image intensity between treatments. Antibodies used include: HSP90 (E289) (Cell Signaling); HSP90 (#2928) (Abcam); HSP90 (ab195575) Abcam; HSP90 3G3 (13495) (Abcam); GRP78 (50b12) (31772) (Cell Signaling); GRP78 (ab191023) Abcam; GRP78 (ab103336) Abcam; GRP78 (N-20) (sc-1050) Santa Cruz; HSP27 (G31) (2402P) Cell Signaling); HSP27 [EP1724Y] (ab62339) Abcam; HSP27 (H-77) (sc-9012) Santa Cruz; HSP27 (LS-C31836) Lifespan science Corp. Other antibodies were as used in prior studies by the laboratory. All immunofluorescent images were initially visualized at 75 dpi using an Odyssey infrared imager (Li-Cor, Lincoln, NE), then processed at 9999 dpi using Adobe Photoshop CS6. For presentation, immunoblots were digitally assessed using the provided Odyssey imager software. Images have their color removed and labeled figures generated in Microsoft PowerPoint.

### Data analysis

Comparison of the effects of various treatments was performed using one-way analysis of variance and a two tailed Student's *t*-test. Statistical examination of *in vivo* animal survival data utilized log rank statistical analyses between the different treatment groups. Differences with a *p*-value of < 0.05 were considered statistically significant. Experiments shown are the means of multiple individual points from multiple experiments (± SEM).

## Abbreviations

ERK: extracellular regulated kinase
MEK: mitogen activated extracellular regulated kinase
PI3K: phosphatidyl inositol 3 kinase
ca: constitutively active
dn: dominant negative
ER: endoplasmic reticulum
mTOR: mammalian target of rapamycin
JAK: Janus Kinase
STAT: Signal Transducers and Activators of Transcription
MAPK: mitogen activated protein kinase
PTEN: phosphatase and tensin homologue on chromosome ten
ROS: reactive oxygen species
CMV: empty vector plasmid or virus
si: small interfering
SCR: scrambled
IP: immunoprecipitation
Ad: adenovirus
VEH: vehicle
